# Pruritus: Progress toward Pathogenesis and Treatment

**DOI:** 10.1155/2018/9625936

**Published:** 2018-04-11

**Authors:** Jing Song, Dehai Xian, Lingyu Yang, Xia Xiong, Rui Lai, Jianqiao Zhong

**Affiliations:** ^1^Department of Dermatology, The Affiliated Hospital of Southwest Medical University, Luzhou 646000, China; ^2^Department of Anatomy, Southwest Medical University, Luzhou 646000, China

## Abstract

Pruritus, the most common cutaneous symptom, is widely seen in many skin complaints. It is an uncomfortable feeling on the skin and sometimes impairs patients' quality of life. At present, the specific mechanism of pruritus still remains unclear. Antihistamines, which are usually used to relieve pruritus, ineffectively work in some patients with itching. Recent evidence has suggested that, apart from histamine, many mediators and signaling pathways are involved in the pathogenesis of pruritus. Various therapeutic options for itching correspondingly have been developed. In this review, we summarize the updated pathogenesis and therapeutic strategies for pruritus.

## 1. Introduction

Pruritus or itching is an unpleasant feeling that causes a desire to scratch, which negatively affects psychological and physical aspects of the life [1]. It is the most common symptom of skin diseases, sometimes trifling or light and sometimes intolerable. It is also the most common reasons for patient to consult dermatologist [2]. The pruritus may exist continuously or occur intermittently. Its site may be local or generalized. Itching is primarily associated with the free teleneuron which distributes in the superficial layers of the epidermis. The most of itching-related skin diseases are contact dermatitis, eczema, urticaria, neurodermatitis, prurigo, and cutaneous pruritus [3]. In addition, the pruritus may emerge from systemic diseases including inflammatory diseases, metabolic diseases, infection, neurologic disorders, endocrine diseases, psychiatric disorders, and cancer [4].

It is generally considered that the cause of itching is extremely complicated and many factors are involved in itching including internal and external factors. The intrinsic factors may be related to chronic infection, block of blood circulation, change of endocrine and metabolism, hereditary tendency to allergies, and so on, while the extrinsic ones are more complex and changeable, consisting of food, inhaled substances, chemical materials, animal hair and fur skin, and so on [5].

Until now, the exact pathogenesis of pruritus remains unknown. Previously, it was thought that histamine mediator was primarily involved in the attack of pruritus [6]. However, recent reports show that some mediators, such as 5-hydroxy tryptamine (5-HT), proteases, opioid peptide, and peptides, play crucial role in the mechanism of itching [7, 8]. Besides, signaling pathways have important effects on it. Accordingly, phototherapy, topical medication, systemic treatment, and traditional Chinese medicine are developed to pave the way for the relief of pruritus [9].

## 2. Clinical Classification of Pruritus

Pruritus, one of the distressing symptoms, covers a variety of clinic complaints containing dermatologic, neurologic, systemic, and psychiatric diseases [10]. In most cases, the origin of pruritus is in the skin or/and the nervous system. Many mechanisms are implicated in the itching [11]. According to the peripheral and central nervous systemic mechanisms, pruritus is divided into the following categories [3, 12, 13].

### 2.1. Skin-Derived Pruritus

Skin-derived itching originates from the skin, which is caused by inflammation, dryness, or damage of the skin. It is produced and irritated by the conduction of C nerve fiber. Some typical diseases, such as urticaria, scabies, and insect bite dermatitis, belong to this category [14–16].

### 2.2. Neuropathic Pruritus

Neuropathic pruritus is associated with pathological alterations in the afferent pathway of sensory nerve fibers. Its coverage is limited to a certain point. Postherpetic neuralgia, for example, is usually accompanied by itching [17–19].

### 2.3. Neurogenic Pruritus

Neurogenic pruritus is derived from the central nervous system, in which itch is produced by the induction and transmission of mediators and receptors without nerve damage. Bile stasis itching, for instance, is caused by opioid peptides acting on the *µ*-opioid receptor [20, 21].

### 2.4. Psychogenic Pruritus

Psychogenic pruritus is a functional itch disorder caused by psychologic factors (some irritating factors, skin dryness, etc.) and psychiatric abnormalities. Parasitic phobia is a common disorder characterized by psychogenic pruritus [22, 23].

### 2.5. Mixed Pruritus

Mixed pruritus is caused by multiple factors and mediated by two or more mechanisms. For example, atopic dermatitis (AD) is a typical disease involving skin derived itching and neurogenic pruritus [24].

## 3. Possible Mechanisms of Pruritus

Although the exact mechanism of itching has not been completely clarified, current studies indicate that some mediators are key contributors to the elicitation and aggravation of pruritus [8]. These mediators play different roles in different itchy conditions. Moreover, it has been proved that signaling pathways and neurotransmitters are also responsible for itch sensation. Thus, the related mechanisms are elaborated in detail as follows [25].

### 3.1. Mechanisms of Mediator-Related Pruritus

Mediator-related pruritus implies that itching is associated with the mediation of mediators including histamine, 5-hydroxy tryptamine, proteases, opioid peptide, peptides, and eicosanoids [26]. There are different mediators involved in the occurrence of pruritus at different stages. It has been found that a variety of mediators, apart from histamine, have much effects on the skin, mainly participating in the occurrence and development of itching [27].

#### 3.1.1. Amines

*(1) Histamine*. Histamine is a chemical medium stored in the basophilic leukocyte and mast cells. When these cells are activated by immune and nonimmune factors, histamine is induced to release [28, 29]. Its receptors belong to the members of the G protein-coupled receptors (GPCR), in which H1 and H4 receptors (H1R and H4R) play important roles in the appearance of pruritus. Previously, it was considered that histamine dominated the development of pruritus via binding to H1R and activating phospholipase C*β*3 (PLC*β*3) and phospholipase A2 (PLA2) [30–33]. Meanwhile, Bell et al. have demonstrated that histamine could increase the calcium influx in the axon terminals of the spinal cord neurons by activating transient receptor vanilloid 1 (TRPV1) receptor and then promote a series of intracellular signal activation and ultimately lead to itching generation [31]. It is currently confirmed that, however, other mediators are greatly important in pruritus occurrence.

*(2) Serotonin/5-HT*. Serotonin or 5-HT in the skin is derived from mast cells, which may induce pruritus through the peripheral and central nervous mediation. At the periphery, it indirectly facilities itching generation by encouraging mast cells to release histamine; at the center, however, it acts as an itchy mediator to produce the pruritus through opioids participation [34, 35].

#### 3.1.2. Proteases

Proteases perform as any enzyme about proteolysis, which are involved in diverse physiological reactions [36]. It is believed proteases are extremely important substances in causing histamine-independent pruritus. Recent studies have demonstrated that proteases play a crucial role in itching attack by combining to GPCR called proteases-activated receptors (PARs), especially PAR2 and PAR4 [37–40].

#### 3.1.3. Cytokines-Interleukins

Interleukins (ILs) are a group of cytokines containing secreted proteins and signal molecules, which were first discovered to be expressed by leukocytes [41]. Some ILs serve as itchy mediators to trigger and exacerbate pruritus. IL-2 and IL-6 are the typical histamine-dependent mediators of pruritus. In cutaneous T-cell lymphoma, for example, IL-3, IL-4, IL-6, and IL-10 synthesized by T-cells promote the secretion of Th2 cytokines particularly IL-6 [42, 43].

#### 3.1.4. Peptides

*(1) Bradykinin*. Bradykinin belongs to an active peptide of the kinin group of proteins. It is a potent inflammatory mediator and endothelium-dependent vasodilator, which contribute to the production of inflammatory reaction and the dilation of blood vessels [44]. The receptors of bradykinin comprise receptor B1 (B1R) and receptor B2 (B2R) belonging to the members of GPCR family. By combining with its receptors, bradykinin initiates and induces a variety of physiological and pathological reaction [45]. In their study, Liu et al. confirmed that B1R was a pivotal factor to facilitate the chronic incurable itching in a diphenylcyclopropenone-treated chronic inflammation mice model [46].

*(2) Substance P*. Substance P (SP) is a neuropeptide widely distributed in the central and peripheral nervous system [47]. After stimulation, SP releases from sensory nerve endings and conveys the signal to center nerves by binding to the NKl receptor (NKR1) [48]. SP works as a messenger in transmission of signals from terminal neurotransmitters and mast cells. However, Andoh et al. recently found that the scratching behavior of mice after intradermal injection of SP was few of connection with mast cells [49].

*(3) Calcitonin Gene Related Peptide*. Calcitonin gene related peptide (CGRP), a member of the calcitonin family of peptides, is produced in both peripheral and central neurons and secreted by peptidergic somatosensory neurons [50]. Its effect on the transmission of itching signals was ever controversial. At present, it is deemed that CGRP plays a regulatory role in the signal transduction of itching through binding to its receptors called calcitonin receptor-like receptor (CALCRL) and a receptor activity-modifying protein (RAMP1) [51]. Moreover, recent studies have reported that prurigo nodularis, a typical itchy disorder with intensive pruritus, is closely associated with the increased dermal levels of CGRP and SP [52].

*(4) Neurotrophin*. Neurotrophin is a large family of physiological activators promoting the growth, differentiation, and maintenance of neurons [53]. It primarily contains nerve growth factor (NGF), brain-derived neurotrophic factor (BDNF), neurotrophic factors-3 (NT-3), and neurotrophic factors-4 (NT-4). Related reports have demonstrated that NGF levels in the itchy lesions of AD and psoriasis significantly increased and correlated with the severity of diseases; NGF, at the same time, upregulated the expression of sensory neuropeptides, which may induce the release of TRPV1, elicit the degranulation of mast cells, and result in pruritus [54–57].

*(5) Opioid Peptides*. Opioid peptides have peripheral and central itchy effects. They effectively work by activation of *μ*-receptor and inhibition of *κ*-receptor in the central nervous system. *μ*-receptor is the major functional receptor for itching production, but *κ*-receptor does the opposite. At the periphery, on the other side, morphine induces pruritus generation by eliciting the degranulation of mast cells. Studies further confirmed that all of these opioid peptides could cause itching after intrathecal administration [58–62].

#### 3.1.5. Phospholipid Metabolites

*(1) Cannabinoids*. Cannabinoids (CB) belong to the derivatives of arachidonic acid, the receptors of which contain CBl receptor and CB2 receptor. CBl receptor is distributed in the central nervous system, while CB2 receptor is distributed in the peripheral tissues [63]. In animal studies, it was found that CB by binding to their receptor could induce the release of 13- endorphins, further to relieve pain and alleviate histamine-induced itching [64]. These results indicate that CB may be involved in the regulation of pain and pruritus.

*(2) Eicosanoids*. Eicosanoids, as signaling molecules, are produced by enzymatic or nonenzymatic oxidation of arachidonic acid [65, 66]. They are vital to diverse physiological and pathological situations, such as regulating cell growth, controlling inflammation, and inhibiting immune responses. There are multiple subfamilies of eicosanoids, consisting of leukotrienes (LTs), prostaglandins, resolvins, lipoxins, eoxins, and thromboxanes [65]. LTs, most prominently, are important regulators in the modulation of pruritus [67]. Andoh et al. discovered that scratching behavior of mice could be induced after the injection of LTB4 into mice skin [68]. Besides, it was found that the levels of LTB4 significantly elevated in AD and psoriatic lesions which were usually accompanied with pruritus.

*(3) Platelet-Activating Factor*. Platelet-activating factor (PAF) has a variety of physiological and pathophysiological effects, which acts as an important mediator and activator in anaphylaxis, inflammation, platelet aggregation and degranulation, and leukocyte chemotaxis. Normally, PAF is produced in low quantities by various cells (e.g., platelets, neutrophils, macrophages, endothelial cells, and monocytes), but it emerges in larger quantities from inflammatory cells in response to specific stimulator [69]. Through specific receptors and a series of signal transduction systems, PAF works to induce diverse biochemical responses. It has been demonstrated that PAF initially evoke an inflammatory response in allergic reactions in the skin of mammals and humans [70].

The primary mediators and their receptors as well as the corresponding medicine are summarized in Table 1.

### 3.2. Mechanisms of Signaling Pathway-Mediated Pruritus

With important progress in knowledge of itch signaling, the pathogenesis of pruritus to some extent becomes clear. Currently, two signal pathways of itching have been identified. One is histamine-dependent (histaminergic) signaling pathway; another is histamine-independent (nonhistaminergic) signaling pathway [71]. In addition, itching can be produced in the central nervous system without relying on peripheral stimulation.

#### 3.2.1. Histamine-Dependent Signaling Pathway

The itchy receptors exist in sensory nerve endings located in the epidermal-dermal connection [28]. These receptors can be combined with the specific mediators mainly involving histamine, 5-HT, SP, and prostaglandins [64, 72]. As members of GPCR, four receptors of histamine (H1~4R) have been confirmed. H1R is a chief receptor involved in itch sensation, which may be activated by coupling with Gq proteins and evoking PLC [30–33]. H1R activation enhances calcium levels and irritates lipoxygenase (LOX) and PLA2. By activation of TRPV1, H1R facilitate scratching response to histamine [30–33]. PLC*β*3, meanwhile, is critical for mediating histamine-induced scratching behavior through H1R in dorsal root ganglion (DRG) neurons [30]. Moreover, the signaling pathway of PLC*β*3 is essential to 5-HT-evoked scratching. These two major signaling transduction pathways are drawn into itch depending on histamine through DRG neuronal mechanism [11].

**Figure 1:**
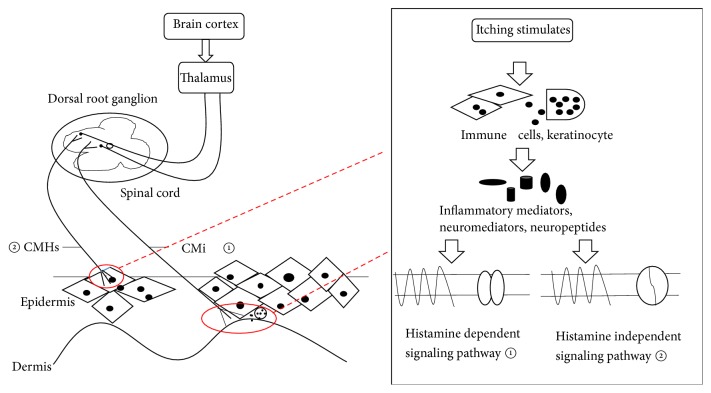
*The possible mechanisms and neurological pathways of pruritus*. Itch stimuli initially induce cells (e.g., immune cells and keratinocyte) in the skin to release many itchy mediators including inflammatory mediators, neuromediators, and neuropeptides. Subsequently, these mediators bind to their receptors, further resulting in the activation of itch-specific sensory neurons. The itch signals are transferred from mechanically-insensitive C-fibers (CMi) called histamine-dependent (histaminergic) or mechanically-sensitive C-type fibers (CMHs) called histamine-independent (nonhistaminergic) signaling pathway, through the dorsal root ganglion (DRG) of the spinal cord, across the spinothalamic tract to the thalamus, ultimately getting to the cerebral cortex.

Besides, various sensory receptors are specially combined with their corresponding ligands to transmit signals and lead to itching. After stimulation by itchy mediators, specific C fibers convey signals to the dorsal horn of the spinal cord and then through the spinal cord to the lamina nuclei of the thalamus and finally to the cerebral cortex (somatosensory area), further producing itch sensation (Figure 1). These C fibers are scarcely sensitive to mechanical stimuli but only to itchy mediators, which therefore are called mechanically insensitive C-type fibers (CMi) [9, 11]. The nerve endings of CMi mainly distribute in the connection of the epidermis and dermis (Figure 1). Moreover, CMi possess some special characteristics including slow conduction rate, many branches of nerve endings, insensitivity to mechanical stimuli, and high threshold of excitation, which plays crucial roles in itch through histamine-dependent signaling pathway [73].

#### 3.2.2. Histamine-Independent Signaling Pathway

**Figure 2:**
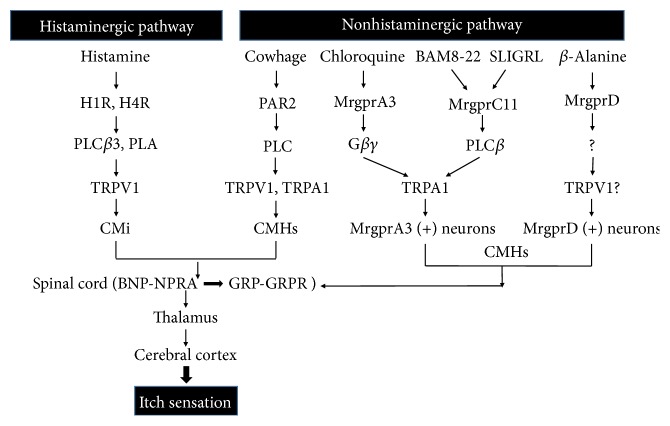
*Schematic illustration of pruritic signaling pathways*. According to different responses to histamine, two signal pathways of itching are covered, namely, histamine-dependent (histaminergic) signaling pathway and histamine-independent (nonhistaminergic) signaling pathway. In the histaminergic pathway, histamine promotes PLC*β*3 and PLC activation by binding to their specific receptors, particularly H1 receptor and H4 receptor. These further induce the activation of downstream target TRPV1. Then, itch signals are transferred to the central nervous system via CMi, which finally lead to itchy sensation. On the other side, many pruritogens exist in the nonhistaminergic pathway, such as cowhage, CQ, BAM8-22, SLIGRL, and *β*-Alanine. Cowhage initially stimulates PAR2, which in turn sensitize PLC. Then the downstream targets including TRPV1 and TRPA1 are activated. Ultimately, itch signals are transferred to the central nervous system via CMHs and itch sensation is produced. At the same time, Mrgprs are linked and activated by CQ, SLIGRL, BAM8-22, and *β*-Alanine, further coupled to G*βγ* or PLC or other; then they promote TRPA1/ TRPV1 activation and Mrgpr-positive neurons detect itch signals; via afferent fibers (CMHs), these signals are sent to the spinal cord and are regulated by GRP-GRPR and BNP-NPRA systems; finally itching sensation is present. PLC*β*3, phospholipase C*β*3; TRPV1, transient receptor potential cation channel V1; TRPA1, transient receptor potential cation channel A1; CMi, mechanically insensitive C-fibers; PAR2, protease-activated receptor; CMHs, mechanically sensitive C-type fibers; BAM8-22, bovine adrenal medulla 8-22 peptide; Mrgprs, Mas-related G protein-coupled receptors; GRP, gastrin-releasing peptide; GRPR, gastrin-releasing peptide receptor; BNP, B-type natriuretic peptide; NPRA, natriuretic peptide receptor A.

Since a major of chronic refractory itch is resistant to antihistamine therapies, it seems that such a chronic pruritus relies on nonhistaminergic mediation. The nonhistaminergic signaling pathway is usually mediated by a class of mechanically sensitive C-type fibers (CMHs) [74]. The nerve endings of CMHs mainly distribute in the epidermis (Figure 1). Itch signals are transferred to the central nervous system via CMHs (Figure 1). CMHs can be stimulated by a tropical leguminous plant---cowhage, which may produce a strong itch sensation when stuck into the skin. Cowhage is a classic nonhistaminergic pruritogen and it induces itching via histamine-independent signaling pathway; thus antihistamine medication ineffectively works [75, 76]. The active ingredient of cowhage is mainly 36KD-cysteine protease called mucunain, which can stimulate PAR2 and PAR4. Present studies suggest that transient receptor potential (TRP) cation channel is the downstream target of the itch signaling pathway, which could be activated by PAR2 [77, 78]. PAR2 initially sensitizes PLC and then stimulates the downstream target including transient receptor potential cation channel V1 (TRPV1) and TRPA1, ultimately leading to itching sensation (Figure 2).

Along with the PARs, there are other kinds of important receptors in mediating histamine-independent itch called Mas-related G protein-coupled receptors (Mrgprs), which specifically distributes in sensory nerves [79]. In 2009, Liu et al. found that the activation of Mrgprs could cause itch sensation, and chloroquine (CQ), another classic nonhistaminergic pruritogen which was previously used in malaria as a drug, contributed to inducing pruritus through histamine-independent signaling pathway by binding to its receptors---MrgprA3 in mice and MrgprX1 in humans [80]. Since human proteins fail to match orthologous pairs to rodent counterparts, Mrgprs in human are called MrgprX1-X4 [79]. Besides, other Mrgprs may be involved in CQ-induced itch because CQ-induced pruritus becomes in part weakening in MrgprA3 cluster-deficient mice [80, 81]. After that, many Mrgprs have been identified as receptors for their corresponding pruritogens; for example, bovine adrenal medulla 8-22 peptide (BAM8-22) was a ligand of MrgprC11 in mice and an activator of human MrgprX1 [82]. Recently, SLIGRL, a protease-cleavage product derived from murine PAR2, was thought to evoke itch by activating MrgprC11 instead of PAR2 [83]. Furthermore, one other Mrgpr linked to nonhistaminergic pruritus is MrgprD, which solely activated by *β*-alanine may elicit itch [84]. Although it is speculated that TRPV1 is maybe involved in the process of *β*-alanine-induced itch, the specific downstream pathway of MrgprD keeps unclear yet. As the critical downstream target of MrgprA3 and MrgprC11, TRPA1 ablation markedly alleviated CQ or BAM8-22-induced scratching response [85]. MrgprA3 is not coupled to PLC but G*βγ* to induce TRPA1 activation, whereas MrgprC11 requires PLC to sensitize TRPA1 [85].

Although the mechanism of Mrgprs mediating itching-related signaling pathways remains elusive, it has confirmed that Mrgprs- and Mrgpr-positive neurons, MrgprA3 in particular, play key roles in mediating chronic pruritus [79, 86]. As we all know, Mrgprs are selectively expressed in primary sensory neurons of the peripheral nervous system. MrgprA3 is specifically expressed in a subset of itch-sensing neurons, called MrgprA3-positive neurons. Other Mrgpr-positive neurons like MrgprD-positive neurons belong to the populations of itch-responsive neurons [79]. MrgprA3-positive neurons are able to be activated by many pruritogens (e.g., chloroquine, BAM8-22, histamine, and cowhage), whereas they fail to respond to *β*-alanine (MrgprD agonist) [87]. Of note, MrgprA3-positive axons innervate the skin, which is responsible for the considerable relief of pruritoceptive itch after MrgprA3-positive ablation [87]. Both Mrgpr-positive neuron populations are stimulated by the substances released from secondary cells like keratinocytes or mast cells, then they detect a variety of itch-inducing molecules through itch receptors on their cutaneous peripheral axons, and finally convey itch signals to the spinal cord via itch-sensing afferent fibers and cause itch sensation (Figure 2) [79, 86].

At present, it has been proved that there exists itch-associated specific central pathways ascending to the brain via the superficial layer of dorsal horn [88]. Typically, gastrin-releasing peptide (GRP), a bombesin-like peptide, is restricted to expressing in lamina I and the outer layer of lamina II; while its receptor, called gastrin-releasing peptide receptor (GRPR), is found to broadly express the central nervous system [89]. When binding to GRPR, GRP can evoke scratching reaction. Likewise, other neural receptors, B-type natriuretic peptide (BNP) receptor in particular, are involved in spinal itch signals transmitting process. BNP, originated from porcine brain, could elicit scratching response via binding to its transmembrane natriuretic peptide receptor A (NPRA) [89, 90]. It has been verified that both GRP-GRPR and BNP-NPRA systems are overwhelmingly implicated in the process of pruritus in the spinal cord [91]. Moreover, BNP-NPRA may function as the upstream of GRP-GRPR system to regulate neurotransmission of itch in the mouse spinal cord [90, 91]. At the beginning, the secondary neurons located in the dorsal horn of the spinal cord and expressing NPRA are activated by glutamate and BNP released from primary sensory neurons [92]. Next, the secondary neurons start to secrete GRP and then activate GRPR of a third neuron in the spinal cord, which ultimately lead to itch sensation [92, 93].

The possible mechanisms of pruritus are described in Figure 1 and the specific signaling pathways of itching are shown in Figure 2.

## 4. Pruritus-Related Clinical Diseases

### 4.1. Pruritus in Dermatoses

#### 4.1.1. AD with Pruritus

Pruritus is outstanding in AD. It is a typical inflammatory skin disease often accompanied with severe and unbearable itching [94]. 10% of children suffer from this disease and it is more popular in adults, especially during pregnancy. Patients with AD tend to have a family history of allergic rhinitis or asthma and be not fully alleviated by antihistamines alone. Apart from histamine, AD is usually caused and mediated by multiple pruritus mediators including neurotransmitters, ILs, and neuropeptides [95].

#### 4.1.2. Psoriasis with Pruritus

Psoriasis is a common, chronic recurrent cutaneous disorder. Although the etiology of psoriasis is unknown, it is currently proposed that many factors, including genetic, immune-based, and environmental factors, are implicated in the pathogenesis of psoriasis [19]. 80% of psoriasis is accompanied with pruritic symptom and itching often lasts for a long time, especially outstanding at night [96]. Most of antipruritic drugs have little effect on this symptom. The itch mechanism remains unclear, but some mediators, such as SP, CGRP, and ILs, have been found in psoriatic lesions; nerve fibers and nerve polypeptides are in addition associated with itching [97].

#### 4.1.3. Herpes Zoster with Pruritus

Although pain is the most popular symptom in herpes zoster (HZ), pruritus commonly emerges from some cured patients with HZ [98]. In their study, Özdemir and Tüzün found 20 of 178 patients with HZ appeared itch sensation and the damaged peripheral nerve located in itch site [99]. The pruritus mechanism may be associated with the damaged nervous system, which limits the symptom to a point in the afferent nerve and causes pruritus through different transmission pathways [15, 16].

### 4.2. Pruritus in Systemic Diseases

#### 4.2.1. Uremic Pruritus

Uremic pruritus (UP) is a frequent and wearisome symptom in patients with end-stage renal disease (ESRD) [100]. The etiology of pruritus caused by renal failure keeps unknown, but it is clarified that antihistamines are ineffective for this disease. At present several theories have been proposed for the development of UP, including imbalanced opiate receptors, abnormal calcium homeostasis, enhanced systemic inflammation, and neuropathic dysregulations [101]. Moreover, recent report has showed that pregabalin is quite useful in the control of treatment-resistant UP by decreasing the calcium influx at the nerve endings and the level of SP, glutamate, and noradrenaline [102]. It is speculated that the pathogenesis of UP may be associated with increased SP and calcium influx.

#### 4.2.2. Cholestatic Pruritus

Pruritus is a common, burdensome, and refractory symptom in patients with cholestasis. The typical itching is extended to the whole body after being localized in the foot joint and palm. At present, the opioid receptor antagonist is the first choice for cholestatic pruritus [103]. Although the pathogenesis of pruritus in cholestasis remains little understood, it is believed that cholestatic pruritus may be mediated by specific neural pathways and pruritogenic factors including opioids, bile acids, and 5-HT [104]. Meanwhile, studies have demonstrated that cholestatic pruritus is possibly related to various mediators such as endogenous opioid peptide, histamine, bile salts, progesterone metabolites, serotonin, and lysophosphatidic acid (LPA) [105, 106].

#### 4.2.3. Diabetic Pruritus

Pruritus is one of the most common signs of diabetes, which may be associated with secondary problems of diabetes, such as candidiasis and xerosis cutis. Clinically, patients with diabetes often feel generalized intractable pruritus without any lesions. Commonly, itching is the first symptoms of diabetes in elder obese patients [107].

#### 4.2.4. Pruritus of Pregnancy

Pruritus is prevalent in pregnancy and distresses the mother [108]. It mainly involves immune and endocrine mechanism, clinically accompanied with increased estrogen and intrahepatic cholestasis [109]. However, itching could rapidly subside after childbirth. Pregnancy pruritus usually initiates in the abdomen, further extending to the thigh, chest, arms, and buttocks. Some specific dermatoses are easily seen in different time during pregnancy, such as prurigo and folliculitis often occurring in second trimester of pregnancy and urticaria in third trimester [110].

#### 4.2.5. Tumorous Pruritus

Stubborn, wide, and inexplicable itching, particularly in the old people, should be alert to the potential possibility of malignant tumor. Pruritus of tumor is either continuous or transient, characterized by circumscribed and generalized manifestations [111]. The pathogenesis of tumorous pruritus remains unclear, presumably contributing to an immune response caused by tumor cells or cell debris [112]. It may be autoimmune causing cells in other parts of the body to be dissolved and release itchy mediators [113].

#### 4.2.6. Senile Pruritus

Senile pruritus is most popular in the elderly population lack of primary lesions. It is a physiological pruritus resulting from skin atrophy, degeneration, skin gland dysfunction, dry skin, and mood swings [114]. This itching most commonly appears in many disorders, such as diabetes mellitus, chronic renal failure, hyperthyroidism or hypothyroidism, cholestasis, parasitic infections, and malignant tumors [115]. In addition, itching is often associated with other diseases such as thyroid diseases, infectious diseases, and anemia; some environmental, physiological, and dietary factors are involved in pruritus including drinking, mood changes, irrational diet structure, bathing frequently, and exposure to allergic substances.

## 5. Strategies for Management of Pruritus

Because of its complicated etiology and pathogenesis, pruritus is often difficult-to-treat and requires interdisciplinary measures. Although not all itching could be successfully controlled by antihistamines, particularly refractory pruritus from malignant tumor and kidney or liver diseases [116], a variety of interdisciplinary therapeutic tools have been developed and applied in clinic during recent decades. These vehicles partly have achieved good results and exhibit promising potential in management of itching.

### 5.1. General Treatment

In general, regular measures should be taken according to the therapeutic principle: finding causative factors, treating original diseases, avoiding all irritating factors, preventing skin dryness, and keeping skin moist [117].

### 5.2. Phototherapy

Ultraviolet B (UVB) has an ability of relieving pruritus through reducing the number of nerve fibers activated by CGRP in peripheral nervous system [118]. Moreover, UVB is extremely effective in control of itching caused by inflammatory skin diseases, uremia, primary cholestasis, globulism, Hodgkin lymphoma, and other systemic diseases [119].

### 5.3. Topical Medication

In clinical practice, many topical medications are frequently used to alleviate the itching. Low-PH cleansing agents, moisturizers, and lubricants are greatly effective in increasing cutaneous irritation [120]. Coolants, at the same time, could transfer the cold to cover the itching via stimulation of the nerve endings [22]; for example, liquid nitrogen is often successfully applied in pruritic dermatoses in our department. Moreover, local anesthetics have better efficacy in moderate pruritus, especially combined with coolants [121]. Owing to their capability of blocking H1 receptors, topical antihistamines are beneficial and usually used for resistance to itching, particularly in the treatment of urticaria and mosquito bites; for example, doxepin is the most useful topical antihistamine [122]. As the most effective topical anti-inflammatory agents, corticosteroids are often used in relief of pruritus from dermatoses caused by itchy mediators, but they always fail to control systemic itching [123]; they should be used only for a short interval, because long-term use would make skin atrophy and dry, sometimes accompanied with corticosteroid-induced acne, rosacea, or perioral dermatitis [124]. Recent immunosuppressants, such as pimecrolimus and tacrolimus, have similar effectiveness to corticosteroid in management of itching, but few significant side effects appear [125]. In addition, optical capsaicin can effectively alleviate pruritus by preventing the synthesis, transmission, and release of SP [126].

### 5.4. Systemic Therapy

Apart from traditional antihistamines, new drugs recently have been developed with further understanding of pruritus mechanisms. These medicines involve many mediator receptor antagonists as follows: opioid receptor antagonists, including naloxone, naltrexone, and nalmefene, have been demonstrated to alleviate pruritus in cholestasis, uraemia, and dermatologic diseases [103, 127–129]. Tricyclic antidepressant, such as doxepin, amitriptyline, trimipramine, and nortriptyline, is effective in resistance to itch of AD [130]. Selective Serotonin Reuptake Inhibitors (SSRI), for example, paroxetine and fluvoxamine, are available to relieve pruritus [131]. Besides, Mirtazapine may attenuate pruritus of patients with ESRD, cholestasis, advanced cancer, and nocturnal itch [132].

In addition to these medicines mentioned above, other drugs, consisting of calcium channel modulator (pregabalin), thalidomide, benzodiazepines (alprazolam), antipsychotic drugs (pimozide), and ondansetron, work well in relief of itching [133, 134].

### 5.5. Chinese Traditional Treatment

Based on the perspective of whole and dialectical therapy, traditional Chinese medicine has great advantages on all kinds of itching. At present, traditional Chinese medicines in management of pruritus mainly cover oral herbal medicine, herbal fumigation, external washing, and acupoint therapy, all of which have obvious effects in relief of pruritus [135]. More importantly, we have demonstrated in our study that tripterygium hypoglaucum hutch, a kind of traditional Chinese medicine, is a good choice for relieving the pruritus of chronic urticaria [136].

## 6. Conclusions

In summary, pruritus clinically covers five categories and extends to a variety of pruritus-related clinical diseases. Although itching mechanism is still unclear, it probably involves various mediators and receptors, the specific nerve fiber, neurotransmitters, and signaling pathways. In spite of poor efficacy in intractable itch with histamines, Hl receptor antagonists, at present, are still widely used as first-line drugs. However, the interaction between H4 and H1 receptors and the development of H4 receptor antagonists should not to be put a high premium. In addition, many molecules are involved in the pathogenesis of itch. Such a complex mechanism indicates that the search for satisfactory vehicles remains a great challenge, and several future strategies for pruritus should be employed such as comprehensive treatment and interdisciplinary measures.

## Figures and Tables

**Table 1:** Mediators, receptors, and drugs about pruritus.

Mediators	Receptors	Drugs
Histamines	Histamine receptors (H1R, H2R, and H4R)	Antihistamines
5-Hydroxy tryptamine (5-HT)	5-HT receptors (5-HT_2_ and 5HT_3_)	Paroxetine, Fluoxetine, Mirtazapine, Ondansetron
Proteases	Proteases-activated receptors (PARs, PAR1–4)	Leupeptin, E6005, E-64, Chymostatin
IL-2, IL-3, IL-4, IL-6, and IL-10	IL-2 and IL-6 receptors	Cyclosporine, Dupilumab, Lebrikizumab
Bradykinin	Bradykinin receptors (B1R and B2R)	Icatibant, Bromelain
Substance-P (SP)	NK receptor (NKR1)	Aprepitant, Fosaprepitant, Casopitant, Vestipitant, Orvepitant, Lanepitant, Dapitant, L-733, 060
Calcitonin gene related peptide (CGRP)	CGRP receptors (CALCRL and RAMP1)	Erenumab, Fremanezumab, Galcanezumab
Opioid peptides	*μ*-receptor, *κ*-receptor	Naloxone, Naltrexone, Nalfurafine
Cannabinoids	Cannabinoid receptors (CB1 and CB2 receptors)	Palmitoylethanolamine (PEA)
Leukotrienes (LTs)	Leukotriene receptors	Zafirlukast, Pranlukast, Montelukast
Platelet-activating factor (PAF)	PAF receptor	Rupatadine, Apafant
